# A critical evaluation of visual proportion of Gleason 4 and maximum cancer core length quantified by histopathologists

**DOI:** 10.1038/s41598-020-73524-z

**Published:** 2020-10-14

**Authors:** Lina Maria Carmona Echeverria, Aiman Haider, Alex Freeman, Urszula Stopka-Farooqui, Avi Rosenfeld, Benjamin S. Simpson, Yipeng Hu, David Hawkes, Hayley Pye, Susan Heavey, Vasilis Stavrinides, Joseph M. Norris, Ahmed El-Shater Bosaily, Cristina Cardona-Barreña, Simon Bott, Louise Brown, Nick Burns-Cox, Tim Dudderidge, Alastair Henderson, Richard Hindley, Richard Kaplan, Alex Kirkham, Robert Oldroyd, Maneesh Ghei, Raj Persad, Shonit Punwani, Derek Rosario, Iqbal Shergill, Mathias Winkler, Hashim U. Ahmed, Mark Emberton, Hayley C. Whitaker

**Affiliations:** 1 Molecular Diagnostics and Therapeutics Group, Division of Surgery and Interventional Science, University College London, Charles Bell House, 43-45 Foley Street, London, W1W 7TS UK; 2 Division of Surgery and Interventional Science, Department of Urology, University College London Hospital, 235 Euston Road, London, NW1 2BU UK; 3 Department of Pathology, University College London Hospital, 60 Whitfield Street, London, W1T4EU UK; 4 Department of Computer Science, Jerusalem College of Technology, Havaad Haleumi 21, Givat Mordechai, 91160 Jerusalem, Israel; 5 Centre for Medical Image Computing, University College London, Charles Bell House, 43-45 Foley Street, London, W1W 7TS UK; 6 Department of Radiology, Royal Free London NHS Foundation Trust, Pond Street, London, NW3 2QG UK; 7 Department Urology, Frimley Park Hospital, Frimley Health NHS Foundation Trust, Portsmouth Road, Camberley, Surrey GU16 7UJ UK; 8 MRC Clinical Trials Unit at UCL, 90 High Holborn, London, WC1V 6LJ UK; 9 Department of Urology, Musgrove Park Hospital, Taunton and Somerset NHS Foundation Trust, Taunton, TA1 5DA UK; 10 Department of Urology, University Hospital Southampton NHS Foundation Trust, Tremona Road, Southampton, Hampshire SO16 6YD UK; 11 Department of Urology, Maidstone and Tunbridge Wells NHS Trust, Hermitage Lane, Tunbridge Wells, ME16 9QQ UK; 12 Department of Urology, Hampshire Hospitals NHS Foundation Trust, Aldermaston Road, Basingstoke, Hampshire RG24 9NA UK; 13 Department of Radiology, UCLH NHS Foundation Trust, University College London Hospital, 235 Euston Road, London, NW1 2BU UK; 14 Public and Patient Representative, 19 Exbury Gardens, West Bridgford, Nottingham, NG2 7SL UK; 15 Department of Urology, Whittington Health NHS Trust, Magdala Avenue, London, N19 5NF UK; 16 Department of Urology, North Bristol NHS Trust, Southmead Road, Westbury-on-Trym, Bristol BS10 5NB UK; 17 Department of Urology, Sheffield Teaching Hospitals NHS Foundation Trust, Royal Hallamshire Hospital, Glossop Road, Sheffield, South Yorkshire S10 2JF UK; 18 Department of Urology, Wrexham Maelor Hospital NHS Trust, Croesnewydd Road, Wrexham, LL13 7TD UK; 19 Department of Urology, Imperial College London, South Kensington Campus, London, SW7 2AZ UK; 20 Imperial Prostate, Division of Surgery, Department of Surgery and Cancer, Faculty of Medicine, Imperial College London, South Kensington Campus, London, SW7 2AZ UK

**Keywords:** Tumour heterogeneity, Urological cancer, Prostate, Medical research, Oncology

## Abstract

Gleason score 7 prostate cancer with a higher proportion of pattern 4 (G4) has been linked to genomic heterogeneity and poorer patient outcome. The current assessment of G4 proportion uses estimation by a pathologist, with a higher proportion of G4 more likely to trigger additional imaging and treatment over active surveillance. This estimation method has been shown to have inter-observer variability. Fifteen patients with Prostate Grade Group (GG) 2 (Gleason 3 + 4) and fifteen patients with GG3 (Gleason 4 + 3) disease were selected from the PROMIS study with 192 haematoxylin and eosin-stained slides scanned. Two experienced uropathologists assessed the maximum cancer core length (MCCL) and G4 proportion using the current standard method (visual estimation) followed by detailed digital manual annotation of each G4 area and measurement of MCCL (planimetric estimation) using freely available software by the same two experts. We aimed to compare visual estimation of G4 and MCCL to a pathologist-driven digital measurement. We show that the visual and digital MCCL measurement differs up to 2 mm in 76.6% (23/30) with a high degree of agreement between the two measurements; Visual gave a median MCCL of 10 ± 2.70 mm (IQR 4, range 5–15 mm) compared to digital of 9.88 ± 3.09 mm (IQR 3.82, range 5.01–15.7 mm) (*p* = 0.64) The visual method for assessing G4 proportion over-estimates in all patients, compared to digital measurements [median 11.2% (IQR 38.75, range 4.7–17.9%) vs 30.4% (IQR 18.37, range 12.9–50.76%)]. The discordance was higher as the amount of G4 increased (Bias 18.71, CI 33.87–48.75, r 0.7, *p* < 0.0001). Further work on assessing actual G4 burden calibrated to clinical outcomes might lead to the use of differing G4 thresholds of significance if the visual estimation is used or by incorporating semi-automated methods for G4 burden measurement.

## Introduction

Gleason pattern 4 (G4) prostate cancer is genetically distinct from Gleason pattern 3 and correlates with worse cancer control outcomes either on active surveillance or following active treatment^1,2^. In 2013 Pierorazio et al., retrospectively reviewed 7850 radical prostatectomy specimens to investigate the short-term biochemical outcome using a prognostic based scoring system called the Prostate Grading Group (GG). By separating the Gleason sum 7 group into 3 + 4 and 4 + 3, the authors found that men with 4 + 3 had worse outcome defined as biochemical recurrence-free survival^3^. These findings were further validated and were subsequently endorsed by the 2014 International Society of Urological Pathology Consensus Conference and the World Health Organization (WHO)^4–6^. Additionally, there is some uncertainty about whether %G4 in 3 + 4 cancers is also relevant to management and outcome^7–9^.

This new classification system calls for improved categorisation of the percentage of G4 (%G4) in Prostate Cancer (PCa) to allow for better risk stratification and inform treatment decisions^7,9–12^. The distinction between Gleason 3 + 4 (GG2) and 4 + 3 (GG3) is made when %G4 falls below or above 50%, respectively, as visually estimated by a uropathologist^5^. Additionally, the maximum amount of cancer in any core (maximum cancer core length, MCCL) has been used as a proxy for tumour volume estimation and can be used to define clinical significance^13,14^.

Most histological prostate cancer burden studies have been performed in radical prostatectomy specimens or on men who have undergone transrectal systematic biopsies. The Prostate MR Imaging Study (PROMIS) includes men who are biopsy naïve whose prostates were systematically sampled every 5 mm providing a unique opportunity to perform an in-depth pathologist-driven annotation and digital analysis of the pathological slides and compare this to the visually-reported %G4 and MCCL^15^.

In this study, we aimed to compare %G4 and MCCL within standard practice, estimated by a pathologist, to a calculated burden from digitally annotated slides by the same pathologists on thirty patients from the PROMIS study with GG2 and GG3 PCa.

## Results

### Comparison between visual and digital MCCL

**Figure 1:**
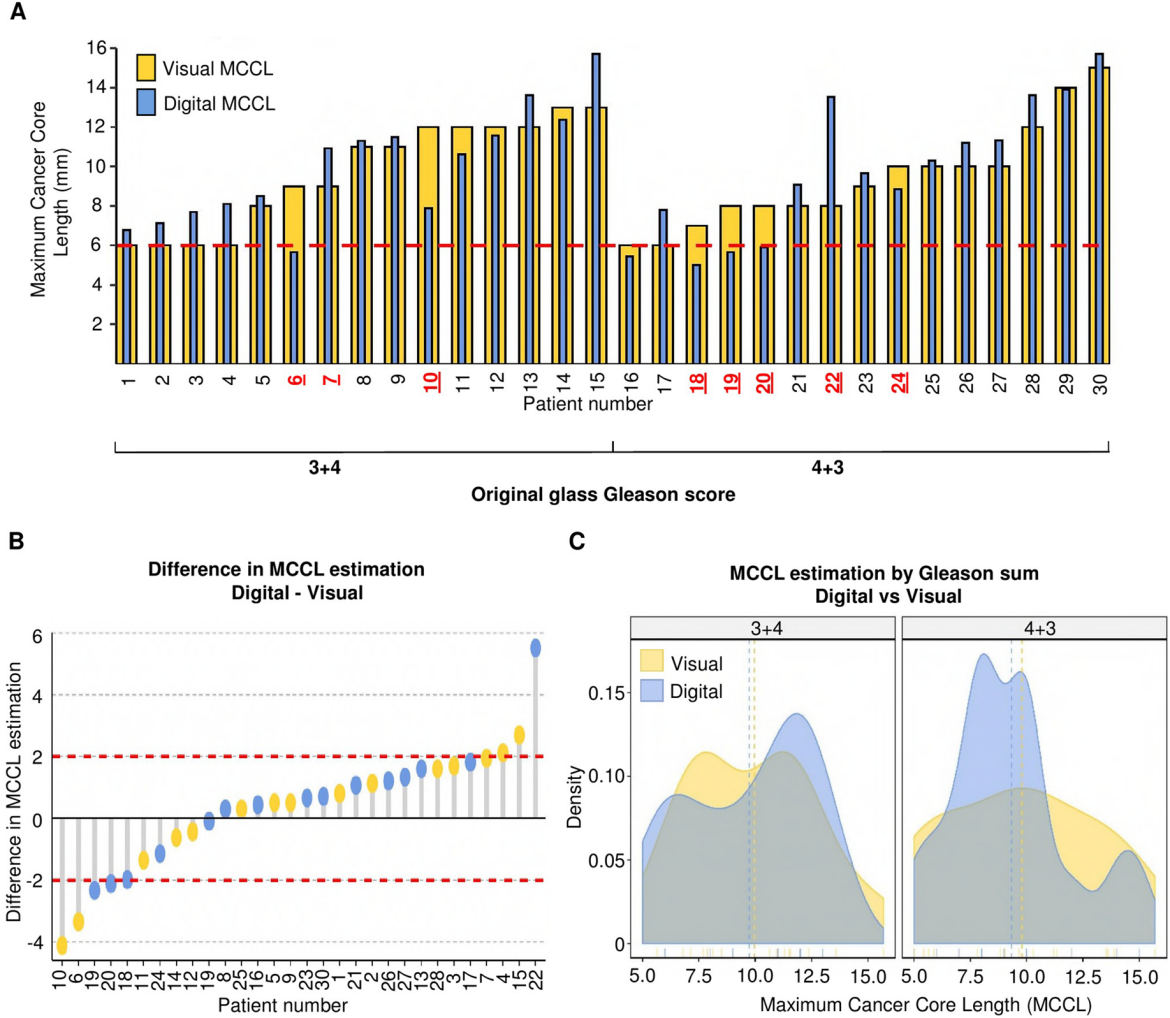
Objective measurement of MCCL and shows a discrepancy with visual measurement and pathologist estimation. (**A**) MCCL difference between visual and digital MCCL shows under-estimation in visual compared to digital MCCL. Bar plot of visual MCCL in yellow and digital MCCL in blue, organised by Gleason score. MCCL is plotted on the y-axis; each patient is plotted on the x-axis. Red dashed lines represent a threshold of 6 mm as the MCCL criterion for significance (PROMIS definition 1). Patients highlighted in red were over or underestimated in the original visual measurement. (**B**) Waterfall plot representing the difference between visual and digital measurements as digital MCCL-visual MCCL by Gleason score (y-axis), patients plotted on the x-axis. Visual Gleason score is represented in yellow for 3 + 4 and blue for 4 + 3. Bars with a negative value represent measurements where the visual MCCL was shorter than the digital MCCL (underestimation). Bars with a positive value represent cases were the visual MCCL was higher than the digital MCCL. The difference in 80% of cases is ± 2 mm (n = 24), red dashed line at − 2 and 2 mm difference. **(C)** Density plots representing the MCCL distribution between visual and digital images by Gleason scores. Y-axis represents the Kernel density estimation. The X-axis contains MCCL values. Visual MCCL score is represented in yellow and blue for the digital measurement. 4 + 3.The mean visual MCCL was 9.53 mm (5–15 mm) and the mean digital MCCL was 9.88 mm (5.01–15.74).

When comparing visual versus digital MCCL, in 23 of the 30 patients the difference was up to ± 2 mm; taking into account the positive and negative values the median difference was 0.58 mm (range − 4.12 to + 5.52 mm, t-test, p = 0.64) (Fig. 1A,B). Seven patients had measurements that differed by ≥ 2 mm between digital and visual estimation. When viewed as a density plot, there was a tendency to overestimate MCCL in the 3 + 4 group and under-estimate in the 4 + 3 group when using the visual method (Fig. 1C). To understand the degree of agreement between the two measurements, a Bland–Altman test was performed^16^. There was no systematic difference (bias) between the visual and digital assessment of MCCL, and there was no correlation between increasing MCCL and the level of disagreement between the two measurements (Supplementary figure S.1).

### Gleason 4

**Figure 2:**
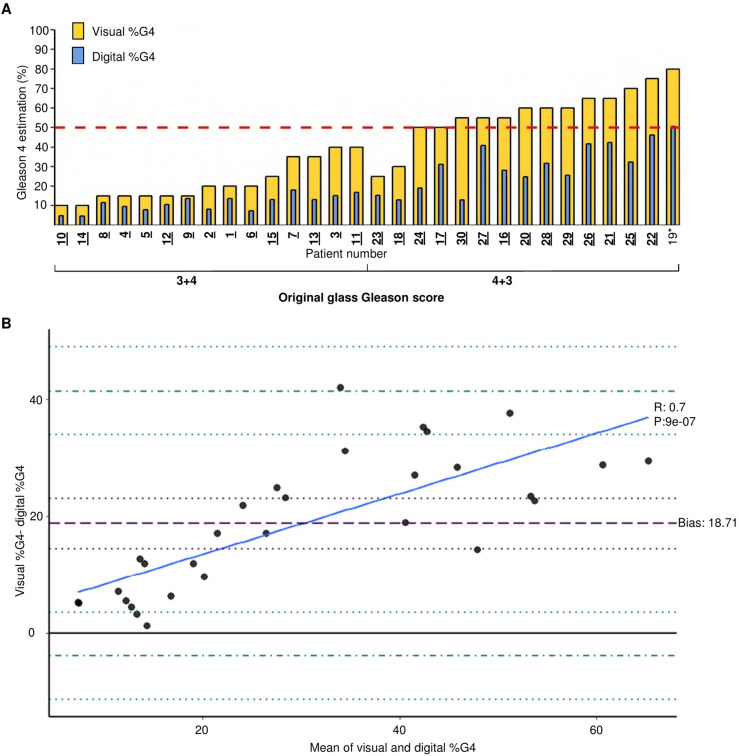
Visual Gleason 4 appraisal overestimates burden of disease. (**A**) Bar plot of the proportion of Gleason 4 estimation average between two uropathologists (yellow) and digital estimation (blue). %G4 is plotted on the y-axis; each patient is plotted on the x-axis. A threshold of 50% g4 for clinical significance is shown as a red dashed line. Patient number on the x-axis is highlighted in bold and underlined if the digital measurement of their %G4 would lead to reclassification based on the digital value. Patient marked with * has ≥ 50% G4 in the digital measurement. (**B**) Bland–Altman plot representing the difference in measurement in the y-axis as visual %G4 – digital %G4. The x-axis represents the mean %G4 measurement of both techniques as (visual %G4 + digital %G4)/2. The bold black line represents complete agreement at 0. The purple dashed line corresponds to the bias at 18.71; the dotted purple line corresponds to the bias confidence interval (33.87–48.75). Dash and dotted blue lines correspond to the upper and lower limit of agreement and confidence intervals are plotted with dotted blue lines. Upper limit of agreement: 41.31 (33.87–48.75), lower limit of agreement: − 3.87 (− 11.31 to 3.56). Regression line is plotted as a continuous blue line.

The visual %G4 overestimated %G4 burden when compared to the digital assessment in all cases (Fig. 2A). The 4 + 3 group had a mean difference of + 26.6% (range 9.6–41.9%) compared to + 10.8% (range 1.3–24.9%) for the 3 + 4 group (t-test, *p* = 1.9 × 10^–5^). The average %G4 in the patients graded 3 + 4 was 11.2% (range 4.7–17.9%) compared to 30.4% (range 12.9–50.6%) in the 4 + 3 group (t-test, *p* < 0.0001). When pathologists were asked to assess the overall Gleason score based on the digital images (visual %G4), two patients were downgraded from their original clinical grading of 4 + 3 to 3 + 4 by both pathologists (See yellow bars of patients 23 and 18 in Fig. 2A).

Using the established 50% G4 threshold to designate a 4 + 3 cancer, and based on the digital %G4 (blue bars), only one patient (number 19 in Fig. 2A) would be classified as 4 + 3. When dividing the digital %G4 into quartiles, two patients in the original 4 + 3 group had less %G4 than the upper quartile of the 3 + 4 group (18 and 30). In other words, these two patients had less %G4 than the men with the highest %G4 compromise in the original 3 + 4 group. Figure 2B shows the Bland–Altman analysis; showing that there was a bias towards overestimation in the visual estimations as all values are located above the line of complete agreement (Complete agreement would result in a zero value). The disagreement was larger when more than 20% of G4 was present (R 0.79, *p* < 0.0001).

**Figure 3:**
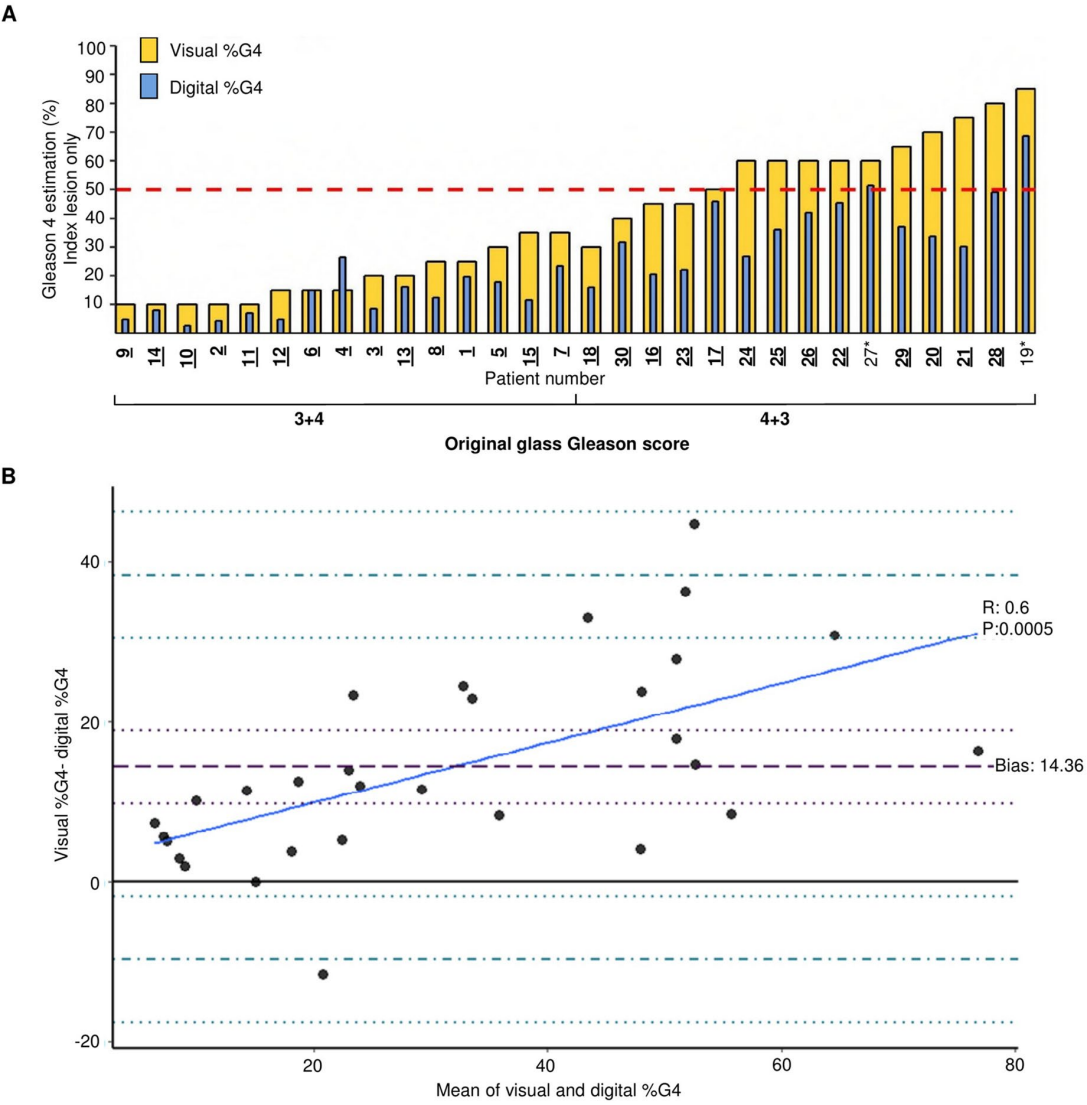
Objective measurement of Gleason 4 burden shows a discrepancy between visual measurement and the digital measurement for the index block. (**A**) Visual %G4 for the index block 30 patients shown in yellow overlaid with digital %G4 in blue. Patients separated by original Gleason grade grouping; 3 + 4 or 4 + 3, and organized by visual %G4. A threshold of 50% G4 for clinical significance is shown as a red dashed line. Patient number on the x-axis highlighted in bold and underlined if the objective measurement of their %G4 would cause reclassification. (**B**) Bland–Altman plot representing the difference in measurement in the y-axis as visual %G4 − digital %G4. The x-axis represents the mean %G4 measurement of both techniques as (visual %G4 + digital %G4)/2. The bold black line represents complete agreement at 0. The purple dashed line corresponds to the bias at 14.36; the dotted purple line corresponds to the bias confidence interval (9.78–18.94). Dash and dotted blue lines correspond to the upper and lower limit of agreement and confidence intervals are plotted with dotted blue lines. Upper limit of agreement: 38.40 (30.49–46.32), lower limit of agreement: − 9.67 (− 17.59 to − 1.76). The regression line is plotted as a continuous blue line.

Examination of the index block (block with the highest Gleason score and MCCL), revealed the same findings as previously seen with all tumour containing cores (Fig. 3A). The visual assessment of digitised images downgraded four patients index block from 4 + 3 to 3 + 4 (patients 18, 30,16, and 23). When examining the digital %G4, only two patients reached the 50% G4 threshold (27 and 19), and so would be the only two patients with 4 + 3 disease based on digital measurement. The Bland–Altman analysis revealed a similar trend to that of the overall %G4 analysis. One measurement had a complete agreement between the digital and visual estimate (Patient 6 in Fig. 3A).One patient had a higher digital estimation compared to the visual estimation (Patient 4 in Fig. 3A). This is represented by the only dot in the negative area of Fig. 3B.The disagreement between measurements increased as the amount of %G4 increased (R 0.6, *p* < 0.0001).

When patients were classified using the clinical significance criteria used in PROMIS in which MCCL and Gleason score were combined to derive definitions 1 (≥ 4 + 3 or ≥ 6 mm) and 2 (≥ 3 + 4 or 4 mm) the digital analysis reclassified four patients’ index block as lower risk^13^. When all blocks were compared using this system, 20 patients had discrepancy between the visual and digital classification, leading to reclassification to higher or lower risk in six and fourteen patients, respectively (Supplementary Figure S2).

## Discussion

We have presented an in-depth analysis of 30 men from the PROMIS trial, to establish the level of agreement between the gold standard visual estimation of MCCL and %G4, compared to digitally annotated images. Limitations to this study include: The presence of cribriform pattern was not recorded separately in this study or included in the final analysis. In addition, the pathologists retrospectively assessed %G4 on annotated images, introducing potential bias in their assessment. Finally, no long term follow up currently exists for the PROMIS study, so we are unable to determine the prognostic significance of our findings.

A threshold of 4 mm and 6 mm has been shown to correlate with 95% of lesions that have a volume higher than 0.2 mL or 0.5 mL, respectively^13^. Demetrios et al*.* found that MCCL greater than 10 mm can predict T3 disease and large tumour volumes with a hazard ratio (HR) of 5.73^14^. Using these thresholds and taking into account the difference in the MCCL measurements, there is a potential impact on the treatment options offered. For instance, patients reclassified as having < 6 mm MCCL could be candidates for active surveillance instead of radical therapy (Patients 6, 18, 19 and 20) (Fig. 1A,B). Interestingly, the visual measurement of men with 3 + 4 disease was more likely to be greater compared to men with 4 + 3 disease (Fig. 1C). Despite these differences, the Bland–Altman analysis showed good concordance between the two measurements; thus, the accuracy of the MCCL is not compromised when a digital tool is used.

In our study, the visual estimation of %G4 differed from the digital one; accurate measurement of the G4 burden has been shown to help risk-stratify patients^9,17^. In a study by de Souza et al*.*, 20% of Gleason 3 + 4 tumours had more extensive G4 disease than the first quartile of 4 + 3 tumours in radical prostatectomy specimens^18^. In 2014, Huang et al*.* found that 45% of men with ≤ 5% of G4 in prostate biopsy had insignificant cancer in radical prostatectomy^7^. Additionally, several papers have shown that tumours with lower %G4 behave closer to GG1 tumours^3,8,9,19–22^.

In this study, we found that visual estimation always overestimated the amount of G4 compared to a digitally calculated %G4. For all of these patients, reclassification of the %G4 would potentially lead to a change in treatment options, and imaging follow up. For example, patient 18 was reclassified after digital assessment and would be downgraded from 4 + 3 of > 6 mm to 3 + 4 of < 6 mm (Fig. 2A). The same was found when we examined the index block only.

Integration of %G4 reporting in biopsies and radical prostatectomy specimens is already recommended^6^. The findings of our study suggest that a re-assessment of %G4 estimation may be required. Reclassification of G4 could lead to a re-evaluation of previously published biomarker and clinical studies and redefine the reference standard for research. The heterogeneity of studies of the prognostic importance of Gleason 3 + 4 disease as compared with Gleason 4 + 3 disease may be a reflection of uncertainty about how much G4 pattern disease is actually shown in specimens and is particularly relevant to treatments such as radiotherapy or ablation where there is no whole mount radical prostatectomy specimen to analyse.

As we move toward the inclusion of digital pathology in standard clinical practice, it will be essential to investigate the differences between human and digital estimation of key pathological parameters and the potential impact this could have on patient care. This will involve adapting the current visual classification to digitally-derived grading. This study does not aim to highlight human error or criticize visual estimation of the pathologists but to encourage the use of technology to improve our understanding of MCCL and G4 burden in prostate cancer, and to seek novel methods to quantify and study the disease. Whilst this type of analysis would be currently challenging to embed directly into clinical practice due to the time taken to contour each region; work is already ongoing to automate this process^23–28^. Identifying relatively overlooked elements, such as %G4, improves the accuracy of the models used in machine learning^29^, as such future algorithms can be trained to specifically identify %G4, rather than GG alone.

Further research is also needed to develop and validate new thresholds of the burden of G4 against large cohorts with medium and long-term cancer control outcomes.

## Materials and methods

### Patients

**Figure 4:**
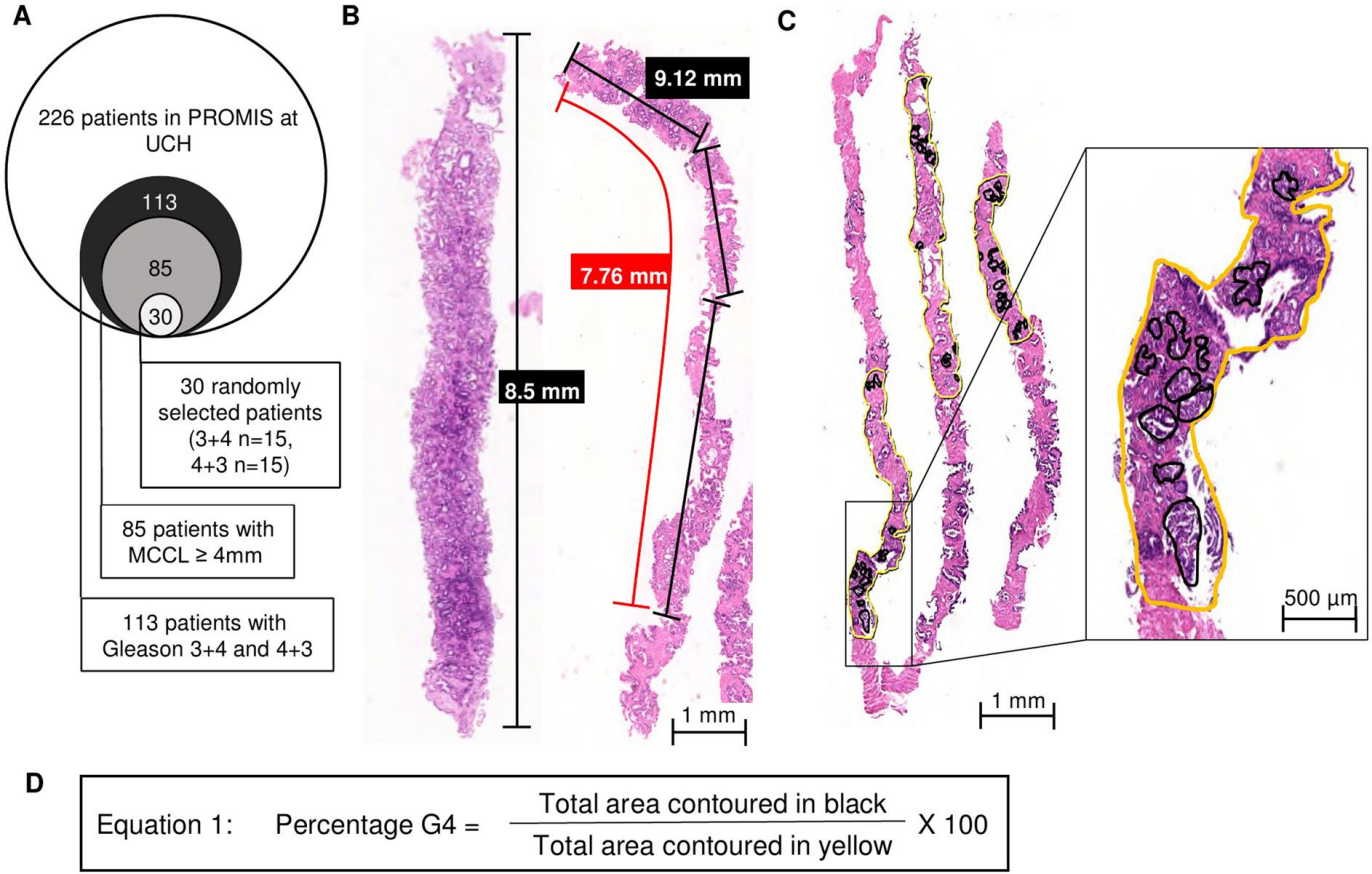
Patient selection and methods of digital manual annotation. (**A**) Euler diagram representing patient selection process for 30 patients for in-depth analysis. (**B**) NDPview2 image of scanned H&E slide of prostate cores from transperineal biopsies, where nuclei are shown in blue, and other structures in pink. From left to right, MCCL measurement in a straight core of 8.5 mm. Approximate visual pathologist measurement marked with a red line (7.76 mm). Following the axis of the core, three measurements in black of 2.53 mm, 2.11 mm and 4.48 mm for a total of 9.12 mm for the digital measurement. (**C**) Three prostate cores, areas with cancer were contoured in yellow, areas with Gleason 4 were contoured in black. Close up of contours shown in black box. Non-contoured areas correspond to benign prostatic tissue. (**D**) Equation used to derive percentage Gleason 4.

Two-hundred and twenty-six patients from University College London Hospital took part in the PROMIS trial. Men underwent 5 mm sampling using a transperineal template mapping procedure. Of 113 men with Gleason 7 PCa, 85 had significant disease (PROMIS definition 1: Gleason 4 + 3 or MCCL ≥ 6 mm). 15 patients with Gleason 3 + 4 and 15 patients with 4 + 3 disease were selected from the 85, using a random number generator (Table 1; Fig. 4A). A mean of 14.2 ± 8.05 cores per patient (IQR 9, range 2–34) were taken. 192 H&E slides from these 30 patients were scanned using a NanoZoomer-SQ digital slide scanner (Hamamatsu).

**Table 1:** Gleason 7 patients in the PROMIS cohort and 30 selected patients for in-depth analysis.

	UCH—PROMIS cohort (4 + 3 or ≥ 6 mm MCCL)		*p* value (3 + 4 vs 4 + 3)	Selected 30 patients		*p* value (3 + 4 vs 4 + 3)
Gleason score	3 + 4	4 + 3		3 + 4	4 + 3	
	n = 67 (78%)	n = 18 (22%)		n = 15 (50%)	n = 15 (50%)	
Age (years)	63 (43–77)	64 (48–79)	0.44*	62 (50–72)	65 (48–79)	0.30*
Prostate volume (cc)	38.34 (16–83)	38.18 (26–55)	0.65**	34 (21–62)	38 (26–55)	0.11**
Presenting PSA (ng/dL)	7.46 (1.30–13)	10.76 (5.7–15)	< 0.0001*	7.60 (4.9–10.1)	10.74 (6.2–15)	0.0005*
PSA density (PSAd)	0.22 (0.06–0.59)	0.29 (0.11–0.53)	0.002**	0.24 (0.10–0.38)	0.29 (0.11–0.53)	0.14*
Likert 2	1 (1.4%)	0		0	0	
Likert 3	8 (11.9%)	3 (16.6%)		1 (6.6%)	0	
Likert 4	21 (31.3%)	3 (16.6%)		6 (40%)	4 (26.6%)	
Likert 5	5 (7.46%)	12 (66.6%)		8 (53.3%)	11 (73.3%)	
Likert NA	4 (5.87%)	0		0	1 (6.6%)	

Table comparing the Gleason 7 patients from University College London (UCH) within the PROMIS study. UCH PROMIS cohort is on the left, selected patients on the right. Number of patients per group by Gleason score in each cohort as n = , percentage in parenthesis. Mean value for age, prostate volume, presenting PSA and PSA density, with range in parenthesis. Age is denoted in years, prostate volume in cubic centimetres (cc), PSA in ng/dL and PSA density calculated as PSA/prostate volume. Likert scores are presented as number of patients and percentage in parenthesis, Likert NA when no Likert score was given. *p-value obtained using an unpaired t-test, **if using Mann–Whitney test.

### Digital scan annotations and data collection

Two experienced UCH uropathologists with 16 years (AF) and 1.5 years’ experience (AH) were involved in this study. The 30 cases included in this study were originally reported by AF as part of the PROMIS trial. The pathologists were blinded to the PROMIS Gleason score; scans were shown randomly and assessed by two experienced uropathologists (AF/AH) using NDP.View 2 software. Each slide was systematically assessed as follows: 1. Each core was numbered from left to right. 2. Length of cancer was measured (Fig. 4B). 3. Areas containing any cancer were contoured in yellow (Fig. 4C). 4. Areas containing G4 were contoured in black (Fig. 4C).

The MCCL was reported prospectively by the pathologists during the trial using the integrated ruler in the microscope; this measurement was assigned as ‘visual’ MCCL. In PROMIS, the MCCL was reported by taking into account intervening benign glands (ISUP) and measuring cancer only. For the purposes of this study, the ISUP measurement was used. The ‘digital’ MCCL was derived as follows: If a core was straight, a single measurement was performed. If there was any curvature, manual sequential measurements were performed along the core axes and combined to give the final measurement.

%G4 was not collected as part of the original trial, pathologists retrospectively visually estimated the %G4 per patient to the closest 10% using the annotated images. This was assigned as ‘visual’ %G4. For digital %G4, the software performs instant area measurements. The resulting area (for each yellow and black contours) was prospectively recorded, and an objective percentage of G4 was calculated as shown in equation 1 (Fig. 4D). This total was assigned as ‘digital’ %G4. A separate analysis of the index block was performed separately. The index block was defined as the block with the highest Gleason score and MCCL in combination with concordance with the index lesion on mpMRI.

### Statistical analysis

Patients were divided according to the original Gleason score from the PROMIS trial into 3 + 4 and 4 + 3. The routinely performed ‘visual’ estimation for both measurements was used as the reference standard for all comparisons. When comparing two groups, meeting normal distribution (Shapiro–Wilk test) and same variances (F-test), a student t-test was applied. Whenever data was not normally distributed a Mann–Whitney test was performed. To quantify the agreement between the two methods, the Bland Altman method was performed. The visual method was used as a standard for comparison; bias was defined as the average of the difference between the two methods. Limits of agreement were calculated at 95% CI. All analyses were made using R: A Language and Environment for Statistical Computing^30^. The Bland–Altman analysis was performed using the blandr package for R^31^.

### Ethical approval

All clinical samples were collected from University College London Hospital NHS Trust patients who had provided informed consent. Ethics committee approval was granted by National Research Ethics Service Committee London (reference 11/LO/0185). Access to biobank samples was obtained [reference (EC/21.16)]. All analyses were performed in accordance with relevant guidelines and regulations.

## Supplementary information

Supplementary Figures.
