# Supplementary material for: A critical evaluation of visual proportion of Gleason 4 and maximum cancer core length quantified by histopathologists
Source: Sci Rep. 2020 Oct 14;10:17177. doi: 10.1038/s41598-020-73524-z (PMC7561724; doi:10.1038/s41598-020-73524-z)
Supplement: Supplementary file 1 — Supplementary Figures. [file 41598_2020_73524_MOESM1_ESM.pdf]

A Critical Evaluation Of Visual Proportion of Gleason 4 and Maximum Cancer Core Length Quantified By Histopathologists.

Running title: Assessment of visual Gleason 4 proportion in PROMIS

Authors

Lina Maria Carmona Echeverria MRCS* linacarmona@nhs.net[1,2],
Aiman Haider FRCPath aiman.haider@nhs.net[3] ,
Alex Freeman FRCPath alex.freeman2@nhs.net[3],
Urszula Stopka-Farooqui MSc u.stopka-farooqui@ucl.ac.uk [1],
Avi Rosenfeld PhD rosenfa@jct.ac.il [4],
Benjamin S. Simpson MSc b.simpson@ucl.ac.uk [1],
Yipeng Hu PhD yipeng.hu@ucl.ac.uk [5],
David Hawkes PhD d.hawkes@ucl.ac.uk [5],
Hayley Pye PhD h.pye@ucl.ac.uk [1],
Susan Heavey PhD s.heavey@ucl.ac.uk [1],
Vasilis Stavrinides MRCS v.stavrinides@ucl.ac.uk [1,2],
Joseph M. Norris MRCS joseph.norris@ucl.ac.uk [1,2],
Ahmed El-Shater Bosaily PhD, FRCR ashater@nhs.net [2,6],
Cristina Cardona Barrena ccardona.b@outlook.com [1],
Simon Bott FRCS simon.bott@nhs.net [7],
Louise Brown PhD l.brown@ucl.ac.uk [8],
Nick Burns-Cox FRCS nick.burns-cox@tst.nhs.uk [9],
Tim Dudderidge FRCS tim.dudderidge@uhs.nhs.uk [10],
Alastair Henderson FRCS alastairhenderson@nhs.net [11],
Richard Hindley FRCS richard.hindley@nhs.net[12],
Richard Kaplan FRCP r.kaplan@ucl.ac.uk [8],
Alex Kirkham FRCR alexkirkham@nhs.net [5,13],
Robert Oldroyd MA re.oldroyd@btopenworld.com [14],
Maneesh Ghei maneesh.ghei@nhs.net [15],
Raj Persad FRCS raj.persad1@nhs.net [16],
Shonit Punwani FRCR shonit.punwani@nhs.net [5,13],
Derek Rosario FRCS derek.rosario@nhs.net [17],
Iqbal Shergill FRCS Iqbal.Shergill@wales.nhs.uk [18],
Mathias Winkler FRCS mathias.winkler@nhs.net [19],
Hashim U. Ahmed FRCS hashim.ahmed@imperial.ac.uk [19,20],
Mark Emberton FRCS m.emberton@ucl.ac.uk [2],
Hayley C.Whitaker hayley.whitaker@ucl.ac.uk [1]

Affiliations:
[1] Molecular Diagnostics and Therapeutics Group, Division of Surgery & Interventional Science. Charles Bell House, 43-45 Foley Street, London. University College London, United Kingdom W1W 7TS.
[2]Department of Urology, Division of Surgery & Interventional Science. 235 Euston road, University College London Hospital, London, United Kingdom. NW1 2BU
[3] Department of Pathology, 60 Whitfield Street, University College London Hospital, London. United Kingdom. W1T4EU.
[4] Department of Computer Science of the Jerusalem College of Technology, Havaad Haleumi 21, Givat Mordechai, Jerusalem, Israel. 91160
[5] Centre for Medical Image Computing. Charles Bell House, 43-45 Foley Street, London. University College London, United Kingdom W1W 7TS.
[6]Department of Radiology, Royal Free London NHS Foundation Trust. Pond Street, London, United Kingdom NW3 2QG.
[7]Department Urology, Frimley Park Hospital, Frimley Health NHS Foundation Trust, Portsmouth Road, Camberley, Surrey, United Kingdom GU16 7UJ.
[8]MRC Clinical Trials Unit at UCL, 90 High Holborn, London, United Kingdom WC1V 6LJ
[9]Department of Urology, Musgrove Park Hospital, Taunton and Somerset NHS Foundation Trust, Taunton, United Kingdom TA1 5DA.
[10]Department of Urology, University Hospital Southampton NHS Foundation Trust, Tremona Road , Southampton, Hampshire, United Kingdom SO16 6YD.
[11]Department of Urology, Maidstone and Tunbridge Wells NHS Trust, Hermitage Lane , Tunbridge Wells, United Kingdom ME16 9QQ.
[12]Department of Urology, Hampshire Hospitals NHS Foundation Trust, Aldermaston Road , Basingstoke, Hampshire, United Kingdom RG24 9NA
[13]Department of Radiology, UCLH NHS Foundation Trust, 235 Euston road, University College London Hospital, London, United Kingdom. NW1 2BU
[14]Public and patient representative,19 Exbury Gardens, West Bridgford, Nottingham, United Kingdom NG2 7SL
[15]Department of Urology, Whittington Health NHS Trust, Magdala Avenue, London, United Kingdom N19 5NF.
[16]Department of Urology, North Bristol NHS Trust, Southmead Road, Westbury-on-Trym, Bristol, United Kingdom BS10 5NB.
[17]Department of Urology, Sheffield Teaching Hospitals NHS Foundation Trust, Royal Hallamshire Hospital, Glossop Road, Sheffield, South Yorkshire, United Kingdom S10 2JF
[18]Department of Urology, Wrexham Maelor Hospital NHS Trust, Croesnewydd Road, Wrexham, United Kingdom LL13 7TD
[19]Department of Urology, Imperial College London, South Kensington Campus, London, United Kingdom SW7 2AZ.
[20]Imperial Prostate, Division of Surgery, Department of Surgery and Cancer, South Kensington Campus, , Faculty of Medicine, Imperial College London, London, United Kingdom SW7 2AZ.

A.

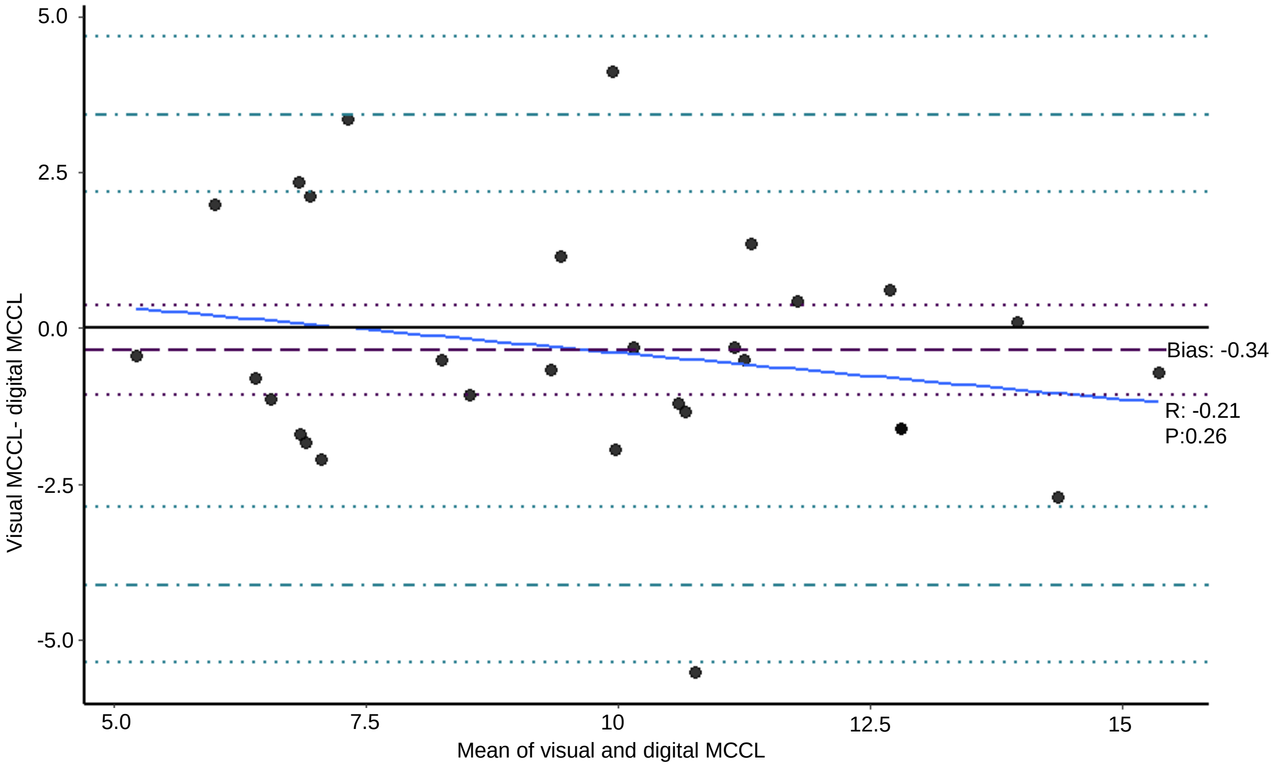

Supplementary figure S1. No significant bias is seen between visual and digital MCCL measurement.

Bland-Altman plot representing the difference in measurement in the y-axis as visual MCCL – digital MCCL. The x-axis represents the mean MCCL measurement of both techniques as (visual MCCL + digital MCCL)/2. The bold black line represents complete agreement at 0. The purple dashed line corresponds to the bias at -0.34. The dotted purple line corresponds to the bias confidence interval (-1.06 to 0.37). Dash and dotted blue lines correspond to the upper and lower limit of agreement and confidence intervals are plotted with dotted blue lines. Upper limit of agreement: 3.43 (2.18 to 4.67), lower limit of agreement: -4.12 (-5.36 to -2.87).

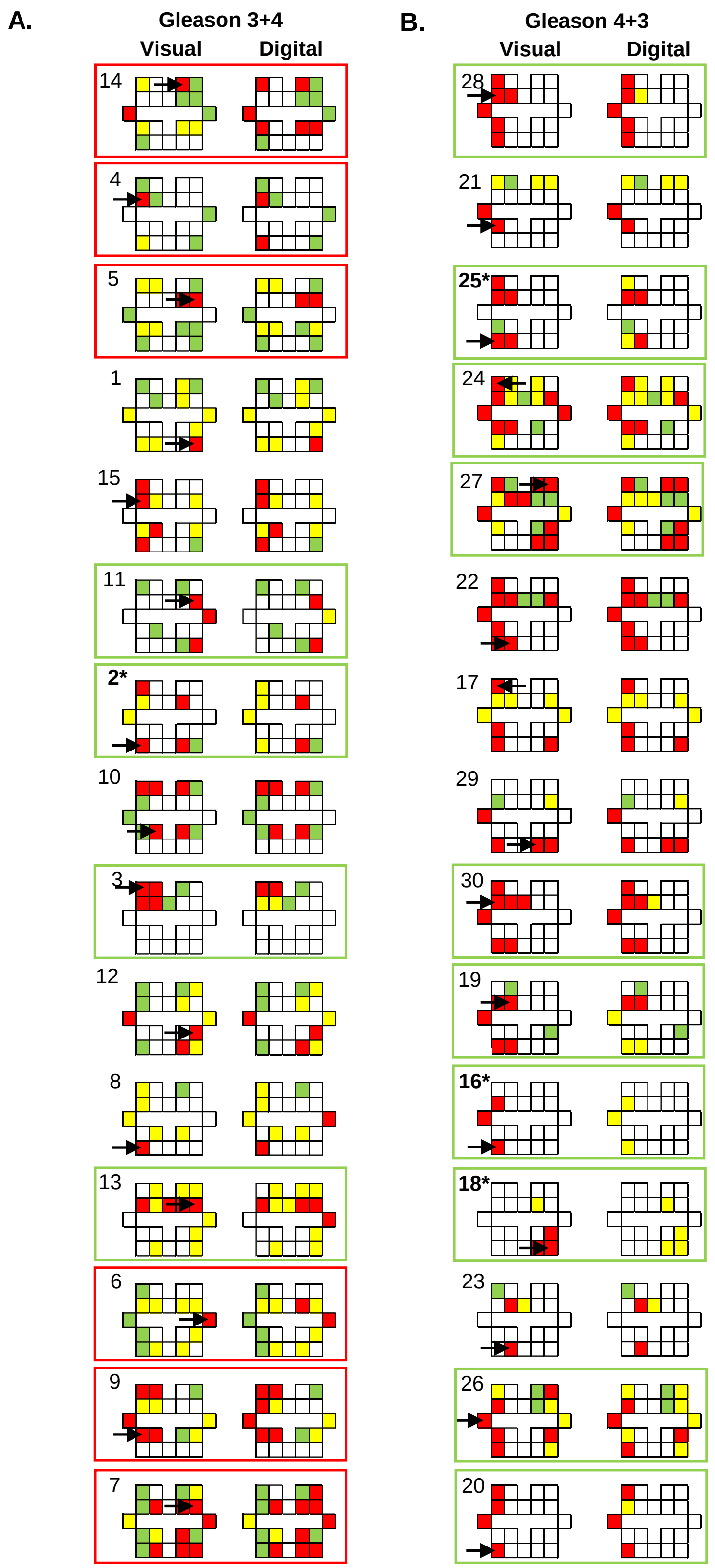

Clinical significance classification

- G 3+3 MCCL ≤ 3 mm
- G 3+4 MCCL 4-5 mm
- G 3+4 MCCL ≥ 4-5 mm
- Index block
- No cancer

Modified Barzell zones

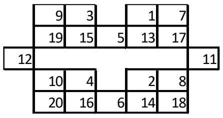

1	Left parasagital anterior apex
2	Left parasagital anterior base
3	Right parasagital anterior apex
4	Right parasagital anterior base
5	Midline apex
6	Midline base
7	Left medial anterior apex
8	Left medial anterior base
9	Right medial anterior apex
10	Right medial anterior base
11	Left lateral
12	Right lateral
13	Left parasagital posterior apex
14	Left parasagital posterior base
15	Right parasagital posterior apex
16	Right parasagital posterior base
17	Left medial posterior apex
18	Left medial posterior base
19	Right medial posterior apex
20	Right medial posterior base

00 ≥ 1 blocks downgraded

00 ≥ 1 blocks upgraded

00* Index block downgraded

Supplementary figure S2. Digital assessment reclassifies patients when using the UCLH traffic light system. Graphical representation of the 20 modified Barzell zones used for transperineal sampling in PROMIS comparing visual and digital assessment, each positive block is denoted by the traffic light system representing the UCH clinical significance classification. Gleason 3+3 and MCCL ≤ 3 mm (green), Gleason 3+4 and/or MCCL 4-5 mm (yellow) and Gleason 4+3 and/or MCCL ≥ 6 mm (red). The index block is highlighted with a black arrow. The table contains the anatomical description of each modified Barzell zone. Patients are separated by original Gleason grade grouping 3+4 (A) or 4+3 (B); the number corresponds to a patient number. * if index block was downgraded. Green boundary box if one or more blocks were downgraded. Red boundary box if one or more blocks were upgraded.
